# Molecular mass screening of mosquitoes for filarial parasites in Germany – re-interpretation of PCR xenomonitoring results would be required

**DOI:** 10.1186/s13071-015-1241-3

**Published:** 2015-12-09

**Authors:** Aleksander Masny, Rusłan Sałamatin

**Affiliations:** Department of Medical Parasitology, National Institute of Public Health – National Institute of Hygiene, Warsaw, Poland; Department of General Biology and Parasitology, Medical University of Warsaw, Warsaw, Poland

**Keywords:** Xenomonitoring, PCR, Filaria, *Dirofilaria*, Infection rate

## Abstract

Comments concerning interpretation of the PCR xenomonitoring results in the article „Molecular detection of *Setaria tundra* (Nematoda: Filarioidea) and an unidentified filarial species in mosquitoes in Germany” Parasites & Vectors 2012, 5:14.

Dear Editor,

In the context of an evaluation of the use of xenomonitoring (sensu [1]) for detecting filarial parasites in Germany we would like to point out that the article by Czajka and colleagues [2] suffers from partially incorrect interpretation, and incomplete description, of PCR results. The performance of *D. repens* DNA detection in mosquitoes using the primers applied in the second round PCRs [3–5], was described neither by Czajka *et al*. [2] nor such performance tests were described in papers published prior to the one by Czajka *et al*. [2] (to the best of our knowledge). Furthermore, only one [6] of the primer pairs used in second round PCRs [3–5] perfectly matches *D. repens* sequences deposited in GenBank [6] – the primer pair 16SOvC and 16SOvB [4, 5]. We asked the authors which primers were used for cytochrome oxidase (COI) gene amplification and the authors informed us that they used, for confirmatory PCRs (second round PCRs), self-designed primers Co1-F and Co1-R that were not mentioned in their article [2].

The authors interpreted every positive result of the screening real time PCR performed on DNA obtained from mosquito pools as positive result of filaria detection. However, the positive results of real time PCR might have been false positive; there were no positive PCR controls described, there were no negative PCR controls described. The sensitivity and the specificity of the PCRs applied in the study were not reported. Considering the above mentioned deficits, the following statement based on PCR results, seems unsupported: “*The absence of* Dirofilaria *spp. or other zoonotic filariae in our sample allows the conclusion that the risk of autochthonous infection in Germany is still very low, although dirofilariasis is emerging and spreading in Europe*” [2]. The results of the screening PCR were used for infection rate calculations however, it was not clearly stated anywhere in the article what was the range of specificity of the screening real time PCR, neither it was tested on positive and negative controls nor its products were sequenced. What authors interpreted as the infection rate was minimum, combined true and false positive result rate of their screening real time PCR – a value with no biological meaning [6]. In the Table one [2] the authors claim that there were 67 positive pools among 666 tested, while on the next page they say that only 24 of the 67 seven samples positive in real time PCR were positive in confirmatory PCR (12S PCR) and 17 were negative (what happened to the remaining 26 samples remains obscure). Of the 24 positive samples 23 were sequenced. What was the result of sequencing of the 23 samples remains unclear to the reader. Thus, the authors themselves admit that not all screening PCR results could be confirmed as filaria positive results by second round PCR, yet they use the number of screening PCR positive results to calculate minimum infection rate of mosquitoes in Table one [2]. Only the samples confirmed to contain filaria by second round PCR and/or sequencing should have been used for minimum infection rate calculations. Furthermore, the authors should publish information which PCR assays were actually used as second round PCRs for COI gene detection [2]. It would be interesting to see the data reanalyzed by the authors which would help to understand better what did molecular mass screening of mosquitoes for filarial parasites in Germany reveal?

## References

[1] Latrofa MS, Montarsi F, Ciocchetta S, Annoscia G, Dantas-Torres F, Ravagnan S (2012). Molecular xenomonitoring of *Dirofilaria immitis* and *Dirofilaria repens* in mosquitoes from north-eastern Italy by real-time PCR coupled with melting curve analysis. Parasit Vectors.

[2] Czajka C, Becker N, Poppert S, Jöst H, Schmidt-Chanasit J, Krüger A (2012). Molecular detection of *Setaria tundra* (Nematoda: Filarioidea) and an unidentified filarial species in mosquitoes in Germany. Parasit Vectors.

[3] Casiraghi M, Anderson TJC, Bandi C, Bazzocchi C, Genchi C (2001). A phylogenetic analysis of filarial nematodes: comparison with the phylogeny of *Wolbachia* endosymbionts. Parasitol.

[4] Krueger A, Fischer P, Morales-Hojas R (2007). Molecular phylogeny of the filaria genus *Onchocerca* with special emphasis on Afrotropical human and bovine parasites. Acta Trop.

[5] Morales-Hojas R, Cheke RA, Post RJ (2006). Molecular systematics of five *Onchocerca* species (Nematoda: Filarioidea) including the human parasite, *O. volvulus*, suggest sympatric speciation. J Helminthol.

[6] Masny A, Sałamatin R, Rozej-Bielicka W, Golab E. Is molecular xenomonitoring of mosquitoes for *Dirofilaria repens* suitable for dirofilariosis surveillance in endemic regions? Parasitol Res. 2015; doi:10.1007/s00436-015-4767-6. 10.1007/s00436-015-4767-6 26490684

